# First-in-class, first-in-human phase I results of targeted agents: Highlights of the 2008 American Society of Clinical Oncology meeting

**DOI:** 10.1186/1756-8722-1-20

**Published:** 2008-10-29

**Authors:** Andrea Molckovsky, Lillian L Siu

**Affiliations:** 1 Division of Medical Oncology and Hematology, Princess Margaret Hospital, University of Toronto, Toronto, Canada

## Abstract

This review summarizes phase I trial results of 11 drugs presented at the American Society of Clinical Oncology meeting held in Chicago IL from May 30 to June 3rd 2008: BMS-663513, CT-322, CVX-045, GDC-0449, GRN163L, LY2181308, PF-00562271, RAV12, RTA 402, XL765, and the survivin vaccine.

## Introduction

This year, a myriad of novel agents were introduced by way of Phase I trials at the American Society of Clinical Oncology (ASCO) meeting, held in Chicago, IL, from May 30 to June 3rd 2008. With the shift of drug development from cytotoxic to targeted mechanisms of action, new and exciting drug classes are being created; over 10 different classes with first-in-human results were identified from this year's meeting alone. These targeted agents, as compared to traditional cytotoxic therapies, may have decreased toxicity and unique pharmacokinetic profiles. Furthermore, armed with pharmacodynamic assays that measure successful inhibition of designated targets, these phase I trial results suggest potential for using biomarkers to help predict and monitor clinical response.

This discussion will focus on phase I results for eleven first-in-class, first-in-human targeted agents: BMS-663513, CT-322, CVX-045, GDC-0449, GRN163L, LY2181308, PF-00562271, RAV12, RTA 402, XL765, and the survivin vaccine. We have limited our discussion to systemic therapies, although phase 1 results for two virus-vector drugs that are injected directly into tumors, OBP-301 and JX-594, were presented at ASCO as well [1,2].

The drugs discussed below are grouped by the cellular location of their intended targets – cell surface, intra-cytoplasmic, or intra-nuclear. Some of these drugs inhibit well-known targets by a novel mechanism, such as the anti-angiogenic adnectins. Other drugs seek to alter the milieu surrounding cancer cells and enhance anti-tumor immunity, such as the antibody to CD-137 (BMS-663513) and the antioxidant inflammation modulator RTA 402. And finally, small-molecule drugs targeting telomerase (GRN163L), survivin (LY2181308 and vaccine), and the hedgehog pathway (GDC-0449) were presented at ASCO this year, marking the culmination of intense pre-clinical research over the past one to two decades for these agents.

All of the drugs under discussion entered phase I trials because of demonstration of anti-tumor effect in vitro and in xenograft animal models. Most of the phase I studies incorporated a standard 3 + 3 dose escalation design, where 3 to 6 patients were treated per dose level [3]. Patient characteristics were typical for phase I clinical trials-all patients had good performance status (ECOG 1 or better), and most patients were heavily pre-treated with standard drug regimens before enrollment. The anti-angiogenic drug trials also excluded patients with intracranial masses, uncontrolled hypertension, and other factors that increased bleeding risk. Dose-limiting toxicities (DLT) were typically defined as grade 3 or worse non-hematological, or grade 4 or worse hematological adverse events, at least possibly related to study drug, occurring within a specified time period after drug delivery, although variations of DLT definitions may exist based on anticipated toxicity from preclinical data. Maximum tolerated dose (MTD) was generally defined as the dose level just below the one at which an unacceptable number of DLTs were encountered (usually > 1/3 or 2/6 of patients), and this dose is typically the recommended phase II dose in most phase I trials. Finally, although evaluation of clinical efficacy is not the purpose of phase I trials, the clinical outcomes for patients enrolled in these trials is of major interest and was presented for most drugs discussed below.

## Drugs that target cell surface moieties

### BMS-663513, a CD-137 antibody

BMS-663513 is a fully humanized monoclonal antibody agonist of CD-137, a tumor necrosis factor (TNF)-receptor that is expressed on the surfaces of activated white blood cells. Stimulation of CD-137 enhances immune response, specifically an anti-tumor immune response, by a variety of mechanisms [4]. Phase I and II data presented by M. Sznol et al. focused initially only on melanoma patients (23 patients in phase I) but expanded to add renal cell carcinoma and ovarian cancer patients (30 enrolled per tumor site in phase II) [5].

The antibody was extremely well tolerated with no MTD reached; only 6% of patients developed grade 3 or higher neutropenia, 15% grade 3 or higher increased liver enzymes. Mild fatigue, rash, pruritis, diarrhea, and fever were observed in up to 15% of patients, with only a few instances of grade 3 or higher fatigue or fever (NB association of fever with neutropenia was not made in the presentation). Toxicity was not related to dose level of drug (ranging from 0.3 mg/kg to 15 mg/kg every 3 weeks).

Partial responses were limited to only 6% of the melanoma patients, although 17% of melanoma patients and 14% of renal cell patients had stable disease at 6 months or longer. Pharmacodynamic studies of blood showed increased levels of activated CD8 cells on day 8 post-treatment, however the increase in CD8 levels, as well as blood levels of other immunologic biomarkers, did not correlate with clinical outcomes.

A phase II clinical trial using BMS-663513 as 2nd line treatment for patients with metastatic melanoma has opened [6]. Presumably since no MTD or recommended phase II dose was found by Sznol et al., this study will be testing different doses of BMS-663513 (ranging from 0.1 to 5 mg/kg every 3 weeks).

### RAV12, antibody to RAAG12

RAV12 is a chimeric IgG1 antibody that targets RAAG12, a carbohydrate moiety attached to cell surface proteins (including many growth factor receptors). RAAG12 is only expressed on epithelial cells lining the gastrointestinal (GI) tract; immunohistochemistry studies reveal diffuse membrane expression of RAAG12 in human GI cancer cells. Binding of RAV12 to RAAG12 induces tumor cell death via oncolysis (direct cell death); in preclinical animal xenograft models only tumor cell lines expressing RAAG12 (at least 10% of cells) demonstrated any response [7].

Lewis et al. presented preliminary phase I data on 53 patients, most of whom had GI cancers (colon, pancreatic, gastroesophageal) and all of whom demonstrated greater than 10% expression of RAAG12 on tumor specimens (whether these were original pathology specimens or biopsies taken at start of trial was not specified) [8]. Toxicity in the form of liver enzyme elevation, abdominal discomfort, and diarrhea was dose-limiting at the highest dose initially tested (1.5 mg/kg weekly). Pharmacokinetic profiling prompted a change from once-weekly dosing to twice and three-times weekly dosing, to minimize peak serum concentrations without compromising steady state levels. After fractionating the dose, Grade 3 or higher liver enzyme elevation was limited to less than 20% of patients, and diarrhea of grade 3 or higher severity was seen in less than 10% of patients. Immunogenicity to the chimeric antibody was seen in 14% of patients, with one anaphylactic reaction documented.

Out of 41 patients evaluated, 7 patients showed some evidence of stable disease, 1 additional patient with pancreatic cancer had stable disease lasting greater than 5 months, and 1 other patient with colorectal cancer had partial response lasting greater than 8 months. A phase II study in combination with chemotherapy, at a dose of 0.75 mg/kg delivered twice weekly, has opened for pancreatic cancer patients and another phase II study is planned for colorectal cancer patients.

### CVX-045, an antiangiogenic fusion molecule

CVX-045 is a fully human monoclonal antibody fused to two thrombospondin-1 (TSP-1) mimetic peptides. TSP-1 is a known inhibitor of angiogenesis [9], and attaching the small TSP-1 peptide to an antibody not only preserves anti-angiogenic properties but greatly extends the half-life [10].

Mendelson et al. presented phase I results for 18 patients with advanced solid tumors treated with CVX-045 in escalating dose cohorts (0.3 up to 18 mg/kg) [10]. Dosing was weekly, with radiologic assessment of response every 8 weeks and pharmacodynamic measurements of tumor vascular permeability via dynamic contrast-enhanced (DCE) MRI before treatment, day 2–4 after first infusion, and day 29 in the higher dose cohorts.

No dose-limiting toxicities were encountered and no immunogenicity to the drug was detected. However two patients experienced serious adverse events attributed to study drug: radiation pneumonitis and bowel obstruction with perforation leading to death. Common mild side effects included fatigue, gastrointestinal upset, dyspnea, headache, dizziness, and anemia. One patient with colorectal cancer demonstrated partial response, and 33% of patients showed stable disease that lasted at least 8 weeks on treatment. DCE-MRI studies demonstrated some changes in blood flow but no change in tumor vascular permeability.

### CT-322, an AdNectin™

Adnectins are a new class of targeted biologics, designed after the ubiquitous protein fibronectin, but with altered binding sites for specific cell-surface targeting. Sweeney et al. presented phase I results for CT-322, a potent vascular endothelial growth factor 2 (VEGFR-2) inhibitor, and the first adnectin to be tested in humans [11]. Thirty-nine patients with solid tumors or non-Hodgkin's lymphoma were enrolled.

Common side effects included proteinuria and hypertension. Grade 3 proteinuria, reversible posterior leucoencephalopathy syndrome, and retinal vascular occlusion were DLTs that lead to an MTD of 2 mg/kg/week; the authors did not specify whether 2 mg/kg/week would be the recommended phase II dose. No infusion reactions and no evidence of antibody formation to the drug were observed. Stable disease in 49% of the 37 patients evaluated was the best response, with a durable response of greater than 12 months in one patient with signet ring carcinoma (unknown primary).

Pharmacodynamic response was measured by levels of vascular endothelial growth factor A (VEGF-A), which has been shown in pre-clinical studies to be increased when VEGFR-2 is blocked [12]. VEGF-A levels did rise within 4 hours after drug injection and remained elevated for at least 4 days post-injection. Higher levels of VEGF-A were seen with increased doses, with a plateau reached at a dose of 1 mg/kg/week.

A phase II clinical trial using CT-322 alone or in combination with irinotecan has opened for patients with recurrent gliobastome multiforme; the dose used in this trial has not been specified [13].

### GDC-0449, a hedgehog pathway antagonist

LoRusso et al presented phase I results of GDC-0449, an oral small molecule inhibitor of Smoothened (SMO) [14]. SMO is a transmembrane protein that localizes to the cell membrane when hedgehog (Hh) ligands (Sonic, Indian, or Desert Hh) bind to cell surface receptor Patched1 (Ptch1). Surface localization of SMO initiates a signaling cascade that leads to activation of glioma-associated (Gli) transcription factors [15]. The hedgehog pathway normally directs organ development during embryogenesis, but can be abnormally activated in cancer cells, particularly in basal cell cancers (BCC) [16].

Three cohorts of patients, totaling 19, with a myriad of solid tumors (containing at least 1 BCC patient per cohort) were enrolled at 3 different dose levels – 150, 270, and 540 mg. Pharmacokinetic data were obtained via a unique dose schedule: first administered dose was day 1, followed by a 2nd dose at day 8 with daily dosing onwards. Half-life of the drug was long, between 10 to 14 days. Maximal drug concentration after a single dose of drug was the same in the 270 and 540 mg cohorts, and steady-state serum levels were the same in all three dose cohorts, indicating pharmacodynamic 'futility' at doses higher than 150 mg with this schedule.

Skin punch biopsies and hair follicles were used for pharmacodynamic analysis. Down-modulation of Gli 1 transcription factor was observed in all skin punch biopsy samples after treatment with GDC-0449.

The drug was extremely well tolerated; drug-related adverse events included grade 2 or less dysguesia in 16% of patients, and grade 3 hyponatremia and fatigue in 10.5% and 5% of patients respectively, with no DLTs. Partial disease response was seen in 2 patients with basal cell carcinoma, and stable disease was observed in another 2 patients with adenocystic carcinoma. The two responding BCC patients were reported to have a very durable response, at 10 months and longer. Phase II studies are now recruiting for GDC-0449 vs. placebo in combination with chemotherapy and bevacizumab for first-line treatment of metastatic colorectal cancer [17], and are being planned for use of GDC-0449 in advanced BCC [14] and as maintenance therapy after 2nd or 3rd remission in ovarian cancer patients [18].

## Drugs whose targets are intra-cytoplasmic

### Survivin inhibitors

Survivin is a member of the inhibitor of apoptosis protein (IAP) family, and has generated interest because of its increased expression in many human cancer cell lines [19]. This year at ASCO, two phase I studies of drugs that target survivin, one through decreasing expression at the mRNA level and the other via vaccination, were presented.

LY2181308 is a new 2'-O-methoxymethyl modified antisense oligonucleotide (ASO) designed to inhibit survivin mRNA expression [20]. Thirty-one patients with various tumors including breast, colon, and melanoma, were enrolled in a phase I study presented by Talbot et al, with LY2181308 given as 3 consecutive daily 3-hour intravenous loading doses followed by weekly maintenance doses [21]. Fever, fatigue, nausea, and elevated partial thrompoplastin times (PTT) were common side effects, while headache was a DLT at the highest dose tested (1000 mg). Pharmacokinetic profiles showed rapid clearance of this intravenous drug after administration, consistent with other second-generation ASOs.

Tumor biopsies were obtained in 23 patients pre-and post-treatment to determine whether survivin expression was decreased; preliminary immunohistochemistry results showed drug penetration in to tumor and decreased survivin levels in 6/12 analyzed pairs of tumor biopsies. Further analysis of survivin gene expression in these samples is planned. Clinical response has so far been limited to stable disease in 10% of patients. A phase II study of LY2181308 in combination with docetaxel chemotherapy in prostate cancer patients has opened [22].

Becker et al presented the phase I/II results of a survivin peptide vaccine, administered to 79 patients, most of whom had melanoma [23]. Three peptides designed for HLA haplotypes A1, A2, and B35 were constructed; patients received 1 to 3 of the peptides depending on haplotype matches. Two dose schedules were tested: three versus six once-weekly injections followed by monthly maintenance injections, with a third cohort receiving the latter regimen after a single 250 mg/m2 dose of cyclophosphamide.

Common low grade side effects included injection site reactions, fever, and painless swelling of the lymph nodes draining the vaccination sites. Immune responses to the drug were observed in 50% of patients, with a trend to higher response in the higher-frequency group. Only patients that demonstrated immune response had any clinical response; of the immune responders, 3 had complete response and 3 had partial response lasting up to 36 months.

### XL765, a dual PI3K and mTOR inhibitor

Phosphatidyl inositol-3-kinase (PI3K) and the mammalian target of rapamycin (mTOR) are enzymes in a common shared pathway – PI3K activates mTOR through another enzyme called AKT. The PI3K/AKT/mTOR pathway is constitutively active in many cancer cells, and plays a key role in cell survival, proliferation, and resistance to chemotherapeutic and targeted agents [24]. PI3K, AKT, and mTOR have been targeted individually by various drugs, but XL765 is the first oral dual PI3K and mTOR inhibitor with Phase I trial results, reported by Papadopoulos et al [25].

Nineteen patients with solid tumors were enrolled and dosing ranged from 15 to 120 mg administered twice daily (bid), with 28-day cycle length. Patients with diabetes or hyperglycemia were excluded from this trial. Transaminitis, diarrhea, anorexia, and fatigue were common mild side effects, with transaminitis and anorexia becoming dose-limiting grade 3/4 toxicities at 120 mg bid; therefore 60 mg bid was chosen as the MTD, although the phase II dose has yet to be decided since additional patients will be enrolled in a once daily dosing schedule.

Pharmacodynamic studies included measurement of plasma insulin levels, since PI3K is also crucial to insulin signaling and its attenuation contributes to type II diabetes [26]. XL765 raised plasma insulin levels in a dose-dependent manner, although grade 1 hyperglycemia was noted in only one patient. Hair samples, skin punch biopsies, and tumor biopsies obtained before and after drug administration demonstrated decreased phosphorylation of various targets in the PI3K pathway, including AKT. Ki67, a marker of proliferation, was also found to be reduced in some tumor biopsy specimens. Best responses to this drug are stable disease lasting at least 3 months in 5 patients, 2 of whom had sustained response for longer than 6 months (one mesothelioma patient and one colon cancer patient).

### PF-00562271, a focal adhesion kinase inhibitor

Focal adhesion kinase (FAK) is a non-receptor protein tyrosine kinase located in the cytoplasm at focal adhesions – sites that link the extracellular matrix to the cytoplasmic cytoskeleton. Not only do FAKs therefore play a pivotal role in cell migration, but they also influence cell survival and are upregulated in a broad spectrum of epithelial cancers [27]. PF-00562271 is an oral reversible inhibitor of FAK, and phase I results for this drug were presented [28].

Sixty-six patients with solid tumors were enrolled and received between 5 mg to 225 mg total daily dose, with scheduling either daily or bid and either fasting or fed, administered in 21-day cycles. Common low-grade side effects included nausea, vomiting, diarrhea, headache, fatigue, dizziness, peripheral neuropathy, anorexia, and edema. Headache and nausea/vomiting were dose-limiting and helped define a recommended phase II dose of 125 mg bid (given with food).

Eleven patients (17%) had stable disease for more than 6 cycles. Positron emission tomography (PET) was used to monitor pharmacodynamic response, with 6 patients showing a 15% or more reduction in uptake of fluorodeoxyglucose (FDG). Moreover, these 6 patients all attained high steady-state serum concentrations of PF-00562271, indicating that PET scanning as a bio-imager may accurately reflect drug bioavailability and potentially clinical response.

## Drugs with intra-nuclear targets

### GRN163L, a telomerase inhibitor

Telomerase maintains telomere length and its over-expression in human cancer cells plays a key role in their immortalization [29]. GRN163L is an oligonucleotide that binds to the RNA active site of telomerase, thereby inhibiting telomerase activity. Ratain et al. presented preliminary toxicity data for patients with various solid tumors in escalating dose cohorts of 0.4 to 4.8 mg/kg per week [30]. Common adverse effects included PTT prolongation, gastrointestinal side effects, fatigue, anemia, GGT elevation, and peripheral neuropathy. One death from unknown causes occurred at 3.2 mg/kg, and thrombocytopenia was a DLT at 4.8 mg/kg. Clinical efficacy data was not available at the time of this report.

### RTA 402, a triterpenoid

RTA 402 is an oral synthetic triterpenoid that inhibits transcription factors NF-κB (nuclear factor-kappa B) and the STAT3 (Signal Transducers and Activators of Transcription protein 3) [31,32]. These transcription factors have gene targets that promote cancer cell proliferation and suppress anti-tumor immunity [33,34]. In addition, RTA 402 induces nuclear erythroid 2 p45 related factor (Nrf-2)-mediated transcription of antioxidant proteins which helps suppress tumor proliferation [35].

Hong et al presented results of a phase I study in which 47 patients, 16 of which had melanoma, were enrolled with RTA 402 dosed daily for 21 consecutive days out of a 28 day cycle [36]. The drug was extremely well tolerated with only 4% or less of patients experiencing grade 3 nausea or fatigue; other side effects included anorexia, diarrhea, and dysguesia. Grade 3 ALT elevation was the DLT at 1300 mg/day, thus 900 mg/day was chosen as the MTD and recommended phase II dose. Pharmacokinetic studies showed that RTA 402 has a long half-life of 39 hours.

Clinical responses were encouraging: of 30 evaluable patients, 40% had stable disease, while one patient with mantle cell lymphoma had a complete response and one with anaplastic thyroid cancer had a partial response. Responses were durable with 50% of responders remaining on drug for 6 months or longer; stable disease responders consisted of patients with melanoma, renal cell, and medullary thyroid cancers.

Biopsies of tumor at days 1 and 21, performed in 5 patients, confirmed inhibition of NF-κB, STAT3 and their target cyclin D1 levels, as well as induction of Nrf2. Interestingly, almost half of the patients who achieved stable disease on drug had peripheral leukocytosis and thrombocytosis, lending weight to the hypothesis that RTA 402 enhances anti-tumor immunity. Phase II studies are being planned in pancreatic cancer (combined with gemcitabine), and in combination with chemotherapy in melanoma patients.

## Discussion

Phase I trials of targeted agents represent the culmination of years of laboratory work and preclinical animal evaluations. Therefore the results are met with excitement and trepidation: excitement for possible clinical benefits and trepidation that the adverse effects of the drug preclude any further development.

Fortunately, the drugs presented this year at ASCO seem to dispel concern regarding toxicity – most were tolerated very well, and only two deaths attributable to the drugs were reported from amongst all eleven of the studies included in this review. In fact, MTDs were not reached for BMS-663513, CVX-045, and GDC-0449, which is unlikely to occur with traditional cytotoxic chemotherapeutics. Choice of appropriate dose for phase II studies therefore relies on other measures; for example the pharmacokinetics of the oral agent GDC-0449 indicated that steady-state plasma concentrations were equal among all doses tested, therefore the lowest was chosen for phase II trials. In contrast, BMX-663513, an antibody whose plasma levels did correlate with increasing dose, but where side effects and response seemed to be independent of dosing, is going forward to phase II clinical trials at different dose levels to help further determine the ideal dose.

The targeted agents presented this year also demonstrate a paradigm shift that is revolutionizing the treatment of cancer – the use of biomarkers to select individual therapies for individual patients. Even from these preliminary phase I trials, where toxicity and dose-finding are the primary goals, interesting pharmacodynamic data were collected. For example, patient selection for the RAV12 antibody was limited to those patients whose tumor specimens demonstrated at least 10% expression of its target RAAG12, although what proportion of total screened gastrointestinal cancer patients showed this level of expression was not presented and would be of interest.

Monitoring of downstream pathways of drug targets was also presented for many of these new agents, again representing potential for predicting clinical response and for proving mechanisms of action. Serum levels of activated CD8 cells and VEGF-A increased after administration of BMS-663513 and CT-322, respectively, as expected by the drugs' mechanisms of action. Tumor biopsies after administration of LY2181308 and RTA 402 confirmed inhibition of their respective targets: survivin and the transcription factors NF-κB and STAT3. Skin punch biopsies were used to illustrate down-regulation of Gli1, a transcription factor activated by SMO, the target of GDC-0449. Hair, skin, and tumor biopsies showed decreased phosphorylation of many products downstream from the PI3K/mTOR pathway inhibited by XL765. DCE-MRI showed modified blood flow within tumors after administration of the anti-angiogenic fusion molecule CVX-045, and PET scanning suggested a correlation between tumor response and steady state serum levels of PF-00562271. None of these phase I trials, except for BMS-663513 and PF-00562271, attempted to correlate pharmacodynamic studies with clinical response, but hopefully phase II studies may expand upon some of these potential predictive markers in more homogeneous patient populations.

Although Phase I studies are not designed to evaluate clinical efficacy these results are of interest. Of the eleven drugs discussed, ten had clinical efficacy data available, and of these ten all showed, at the very least, some stable disease responders. A number of phase II studies have already opened, encompassing such tumor sites as colorectal cancer, melanoma, gliobastome multiforme, and prostate cancer. Table 1 summarizes the important clinical findings of the eleven drugs discussed above.

**Table 1:** Summary of eleven first-in-human drugs presented at this year's ASCO meeting.

**Drug name**	**Target**	**Route**	**Tumor sites enrolled**	**Common low-grade adverse effects**	**Dose-limiting toxicities**	**RPTD**	**Clinical efficacy**	**Phase II studies planned/o pen**
**BMS-663513**	CD-137 agonist	i.v.	Melanoma Renal cell Ovarian	Fatigue Rash/pruritis Diarrhea Fever	Neutropenia Transaminitis	TBD (range from 0.1–5 mg/kg every 3 weeks)	6% PR 15% SD†	Melanoma

**CT-322**	VEGFR-2	i.v.	Misc. solid tumors and NHL	Proteinuria Hypertension	Proteinuria RPLS Retinal vascular occlusion	MTD of 2 mg/kg/week, RPTD not specified	49% SD†	GBM

**CVX-045**	Thrombospondin	i.v.	Misc. solid tumors	Fatigue GI Dyspnea Headache Dizziness	None 1 radiation pneumonitis 1 death (bowel obstruction)	12 mg/kg/week	5% PR 33% SD	TBD

**GDC-0449**	Smoothened	oral	Misc. solid tumors	Dysguesia Hyponatremia Fatigue	None	150 mg daily	11% PR 11% SD	Colorectal Ovarian BCC

**GRN163L**	Telomerase	i.v.	Misc. solid tumors	Prolonged PTT GI Fatigue	Thrombocytopenia One death (unknown cause)	TBD	TBD	TBD

**LY2181308**	Survivin	i.v.	Misc. solid tumors	Fever Fatigue Prolonged PTT Nausea	Headache	750 mg daily for 3 days, then weekly	10% SD	Prostate

**PF-00562271**	FAK	oral	Misc. solid tumors	GI Headache Fatigue Dizziness	Headache Nausea	125 mg twice daily	17% SD	TBD

**RAV12**	RAAG12	i.v.	GI cancers	Diarrhea Abdominal discomfort Transaminitis	Diarrhea Abdominal discomfort Transaminitis	0.75 mg/kg twice weekly	2% PR 20% SD†	Pancreas Colorectal

**RTA 402**	NF-κB and STAT3	oral	Misc. solid tumors and NHL	GI Fatigue Anorexia Dysguesia	ALT elevation	900 mg/day	7% CR + PR 40% SD†	Pancreas Melanoma

**XL765**	PI3K and mTOR	oral	Misc. solid tumors	Transaminitis Diarrhea Anorexia Fatigue	Transaminitis Diarrhea	Possibly 60 mg twice daily	26% SD	TBD

**Survivin vaccine**	Survivin	s.c.	Melanoma	Injection site reactions Fever Lymph node swelling	None	TBD	8% CR+PR	TBD

†Clinical efficacy was not available at time of presentation for all patients enrolled in these studies, therefore these response rates do not represent the whole study cohort and may change in the future

Abbreviations: TBD – to be determined; MTD – maximum tolerated dose; RPTD – recommended phase II dose; CR – complete response; PR – partial response; SD – stable disease; i.v. – intravenous; Misc – miscellaneous; NHL – non-Hodgkin's lymphoma; GBM – gliobastome multiforme; PTT – partial thromboplastin time; s.c. – deep subcutaneous; GI – gastrointestinal (including all 3 of nausea/vomiting/diarrhea); BCC – basal cell carcinoma; RPLS – Reversible posterior leucoencephalopathy syndrome

In summary, phase I trial results for eleven first-in-human, first-in-class targeted drugs hold promise for future clinical applications. Toxicity was acceptable for all the drugs, and clinical efficacy, although premature, shows potential. Pharmacodynamic analyses demonstrate that these targeted agents actually do target the desired pathway of interest, and may be useful for future biomarker applications. Phase II studies are underway for many of these drugs in a broad array of tumor sites and will hopefully translate into meaningful clinical results. Certainly, the area of oncology therapeutics is burgeoning; a recent analysis demonstrated that between the years 2005 and 2007, oncology trials comprised the largest therapeutic area enrolled in the US Clinical Trials database, with the most early phase clinical trials as well [37]. This year's ASCO and its multiple first-in-human agents entering the clinical arena is a further confirmation of this phenomenon.

## Competing interests

AM has no competing interests to declare.

LLS has assumed consultant or advisory roles for: Enzon Pharmaceuticals and Millennium Pharmaceuticals; and has received research funding from: Abraxis Bioscience Inc., Amgen, Bristol-Myers Squibb, Cyclacel Pharmaceuticals, Inc., Novartis Pharmaceuticals, Pfizer, F. Hoffmann-La Roche Limited, Twinstrand Therapeutics.

## Authors' contributions

AM collected and assembled the data, and participated in conception and design, data analysis and interpretation, and manuscript writing. LS participated in conception and design, data analysis and interpretation, and manuscript writing. Both authors read and approved the final manuscript.
